# Blaschko-Linear Lichen Planus Pigmentosus: An Unusual Presentation

**DOI:** 10.7759/cureus.20047

**Published:** 2021-11-30

**Authors:** Madiha Eljazouly, Fatima Zahra Agharbi, Maha Alj, Kenza Oqbani, Soumiya Chiheb

**Affiliations:** 1 Dermatology Unit, Cheikh Khalifa International University Hospital, Mohammed VI University of Health Sciences, Casablanca, MAR; 2 Pathology Unit, Cheikh Khalifa International University Hospital, Mohammed VI University of Health Sciences, Casablanca, MAR

**Keywords:** linear lichen planus, radiotherapy, blashkoid, lichen planus pigmentosus, lichen planus

## Abstract

Lichen planus pigmentosus (LPP), an uncommon variant of lichen planus (LP), is characterized by diffuse hyperpigmented dark brown macules in sun-exposed areas. We report an unusual case of LPP with a blaschkoid distribution in an area of radiotherapy for breast cancer. This description is rarely reported. Its pathogeny is poorly understood and suggests an embryological origin by genetic mosaicism and also discusses the immunomodulatory role of radiotherapy in the disease.

## Introduction

Lichen planus pigmentosus (LPP), an uncommon variant of lichen planus (LP), is characterized by acquired dark brown to gray macular pigmentation located on sun-exposed areas of the face, neck, and flexures, commonly found in dark-skinned patients [1]. It may be diffuse, reticular, blotchy, perifollicular, annular, and less frequently linear [2]. We report a rare case of linear LPP, which follows lines of blaschko in an area of radiotherapy for breast cancer on the trunk without a history of sun exposure.

## Case presentation

**Figure 1:**
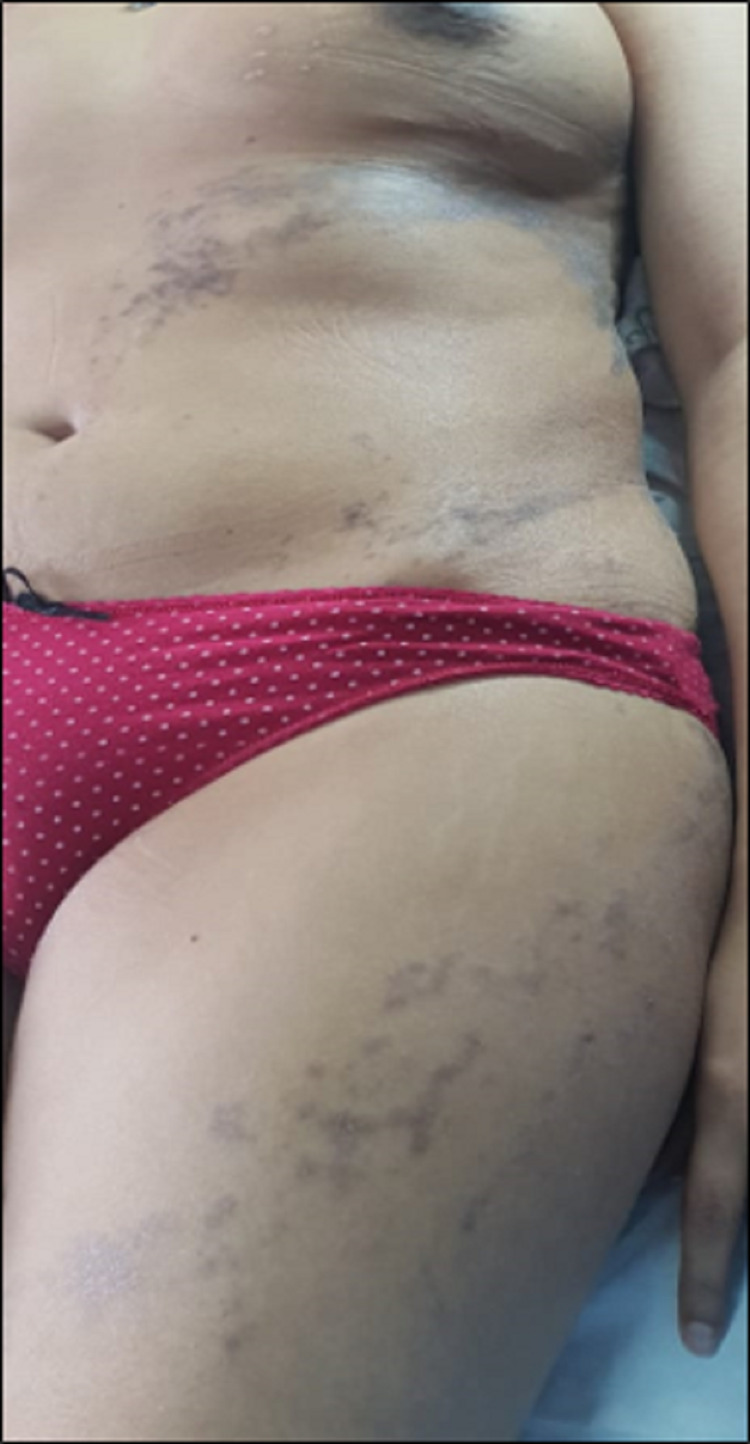
Lichen planus pigmentosus with characteristic hyperpigmented macules and patches following lines of blaschko.

**Figure 2:**
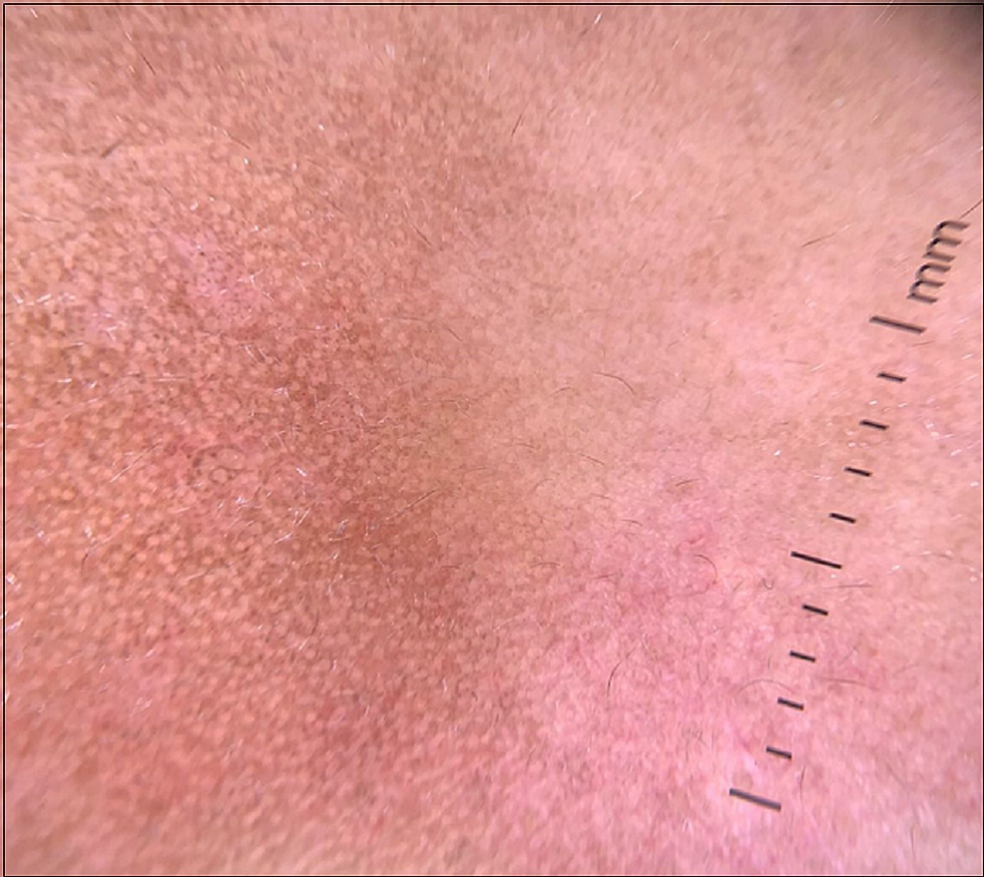
Dermoscopy showing brown dots arranged in a linear and reticular pattern with a few brown and erythematous homogeneous areas.

A 38-year-old woman follow-up for two years for infiltrating ductal carcinoma in the left breast. After conservative surgery, she received adjuvant systemic chemotherapy. Radiation therapy was performed at the tumor site. She presented three months after radiotherapy a purple papular rash on the left side of her trunk extending distally in a linear fashion on her abdomen and upper left thigh. The lesions were poorly delineated and spared the right half of the trunk stopping at the anterior midline (Figure 1). The patient did not have mucosal lesions or nail damage. The eruption was moderately pruritic. Dermoscopy showed brown dots arranged in a reticular pattern with brown and erythematous areas (Figure 2). Different diagnoses were considered such as drug reaction, linear LPP, linear LP, and postinflammatory hyperpigmentation (PIH).

**Figure 3:**
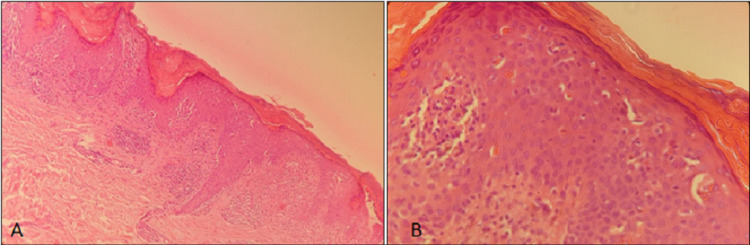
Histological demonstration showing lichenoid inflammatory infiltrate with numerous melanophages (hematoxylin and eosin (A) 10x, (B) 40x).

Histopathological examination showed atrophic epidermis, with lymphohistiocytic infiltrate, and numerous melanophages. These features were suggestive of LPP (Figures 3A, 3B). Breast imaging and hepatitis B and C were negative. The patient was treated with topical corticoids and tacrolimus with the improvement of the lesions and pruritus.

## Discussion

LPP, first described by Bhutani et al., differs clinically from classical LP. It is characterized by macular hyperpigmentation distinct in color ranging from blue, dark brown, grey, and black without pruritus or scalp, nail, or mucosal involvement [3]. It usually affects the sun-exposed areas such as the face, neck, and flexural folds.

Blaschkoid LP has been commonly reported in contrast to the blashkoid LPP. LPP along blaschko’s lines is also known as linear LPP if it affects the limbs or the face. It is considered a zosteriform LPP if the lesions develop in a dermatomal arrangement. It may be unilateral or bilateral, distributed along with one or many Blaschko’s lines. Some authors suggest that linear arrangement according to Blaschko's lines would be due to the presence of an inapparent keratinocyte clone, but genetically modified by mosaicism and reacting specifically to potential triggers such as exogenous immunogenic factors (drugs, pregnancy, sun exposition) or infectious (virus) [4,5]. Radiotherapy is known to have immunomodulatory properties. Exposure to low doses of radiation modulates the function of both macrophages and T lymphocytes and induces a selective enhancement of CD8+ T-cell responses [6,7]. LP after radiation has been described, but not a linear variety, and the previously reported case was believed to be a Koebner response. In our patient, the role of radiotherapy was initially raised because of the localization at the irradiation site and the acquired character of the eruption. However, the extension of the lesions outside the irradiation site, with the average delay rules out the koebner phenomenon and pleads in favor of the immunomodulatory role of the radiotherapy and other factors (stress, drugs) [7,8]. In addition, breast imaging was performed to rule out tumor recurrence and paraneoplastic origin.

Several differential diagnoses can be discussed, erythema dyschromicum perstans (ashy dermatosis), linear epidermal nevus, macular amyloidosis, linear LP, adult blaschkitis, and PIH [8,9]. However, the linear epidermal nevus appears early in life and evolves by very in highly pruritic inflammatory flare-ups and adult Blaschkitis involves papulovesicular lesions with evolving also in inflammatory flare-ups. The histopathological examination allows eliminate other diagnoses by showing characteristically, orthokeratosis, focal basal liquefaction, a sparse perivascular inflammatory infiltrate in the papillary dermis, and pigmentary incontinence [9].

The course of blaschkoid LPP is usually benign but chronic and persistent if treatment is not initiated early. Topical glucocorticoids and tacrolimus are considered first-line therapy with additional skin lightening creams containing hydroquinone and retinoids. Systemic therapies including oral glucocorticoids, dapsone, Isotretinoin, have been recently reported as promising therapy for refractory cases [10].

## Conclusions

Blaschkoid LPP represents a distinct entity of LP whose etiopathogenic remains a mystery suggesting a theory of embryonic mutation of keratinocytes resulting in distinct properties that may render them more susceptible to potential triggers.

Currently, anti-inflammatory treatment for a prolonged period with the avoidance of recognized triggers has been suggested to prevent relapses of blaschkoid LPP and improve pigmentation and the aesthetic appearance of patients as well as their quality of life.
